# Considering How Best to Allocate Limited Resources for Healthcare in Lower-Income Settings–Reflections on Ghanaian Community-Led Data Collection

**DOI:** 10.3389/ijph.2022.1605434

**Published:** 2022-10-31

**Authors:** O. K. Afreh, P. Angwaawie, J. K. E. Attivor, L. A. Boateng, K. Brackstone, M. G. Head, A. K. Manyeh, G. A. A. Vidzro

**Affiliations:** ^1^ Regional Health Directorate, Ghana Health Services, Accra, Ghana; ^2^ Nkwanta South Municipal Health Directorate, Ghana Health Services, Accra, Ghana; ^3^ Clinical Informatics Research Unit, Faculty of Medicine, University of Southampton, Southampton, United Kingdom; ^4^ Institute of Health Research, University of Health and Allied Sciences, Ho, Ghana

**Keywords:** survey research, community health, neglected tropical diseases, Ghana, NTD

As the world begins to transition beyond the urgent phase of the pandemic response, countries are increasingly looking to “mainstream” their COVID-19 management comprising of vaccination and case management into routine healthcare. Access to routine healthcare was severely limited across much of 2020 and 2021, with the greatest impact noted in lower-income settings [1].

Population health undoubtedly has been negatively impacted, and countries are even more unlikely to reach Sustainable Development Goal targets by 2030. Policymakers, therefore, need up-to-date evidence to plan healthcare delivery and direct their limited resources toward priority areas. One effective, but resource-intensive, approach to disease control is Mass Drug Administration (MDA). An MDA is where preventive chemotherapy is used as a strategic approach to treat populations at risk of infection, with medicines being distributed across communities [2]. These form a core component of the 2021–2030 WHO Neglected Tropical Diseases (NTDs) Roadmap [3]. The WHO targets for MDAs typically require greater than 80% uptake to be considered effective with disease control [4]. With a community-wide intervention such as an MDA, local acceptance is vital to ensuring high engagement.

Ghana is a lower-middle income country in West Africa with approximately 30 million people. Like many countries in sub-Saharan Africa, Ghana has used MDAs successfully to control diseases such as onchocerciasis and lymphatic filariasis [4]. During the COVID-19 pandemic, and following WHO guidance, MDAs mostly did not take place in Ghana.

**FIGURE 1:**
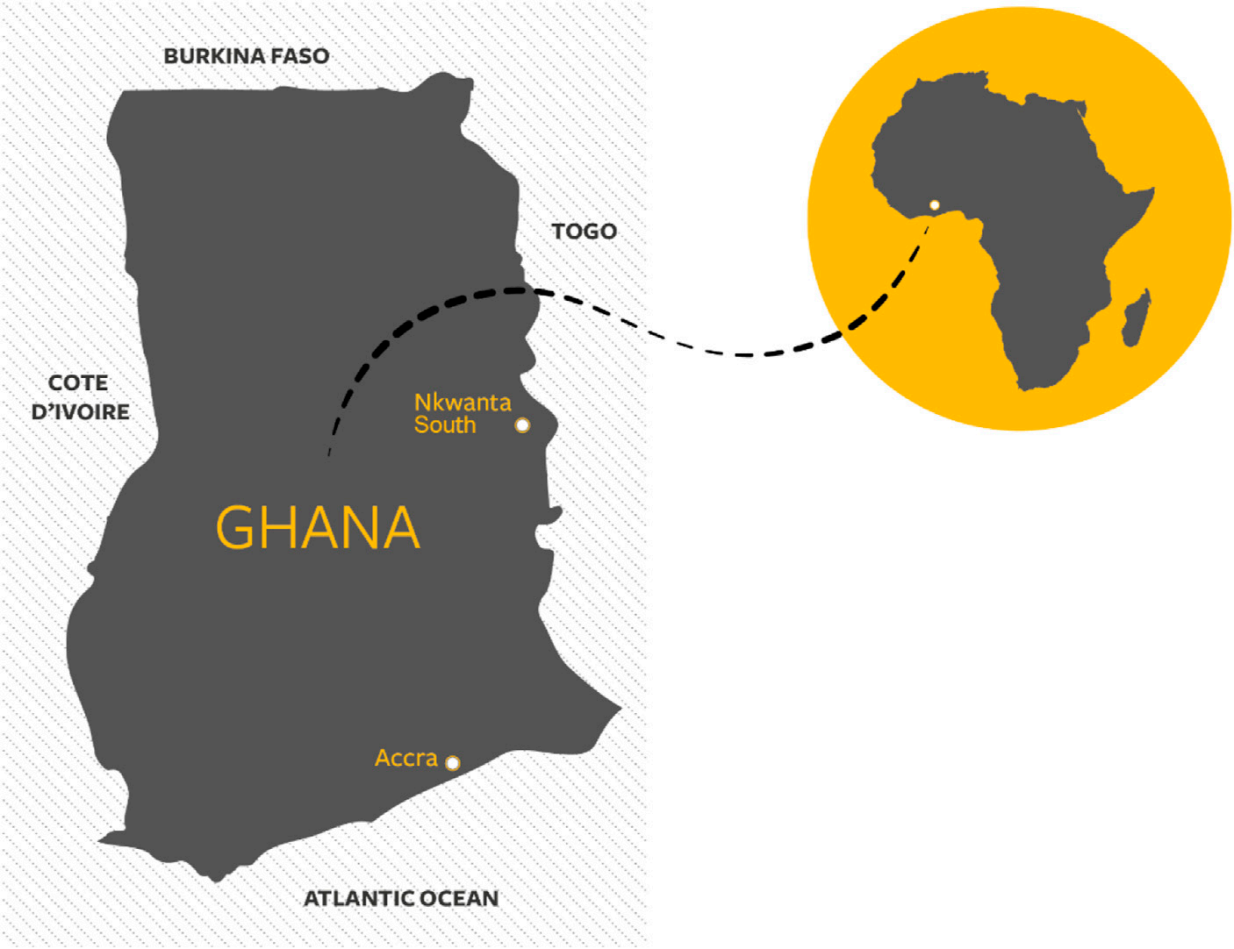
Map illustrating Ghana and the location of Nkwanta South municipality (Oti region, Ghana, 2022).

There are ongoing research partnerships between the Ghana Health Service, the University for Health and Allied Sciences (Ghana), and the University of Southampton (UK). These include the Oti Regional Health Directorate and Nkwanta South Municipality (a rural area close to the Togo border) (Figure 1).

In January 2022, a research survey took place around community viewpoints on the pandemic response and COVID-19 vaccination [5]. Alongside this, a small number of supplementary questions were asked around knowledge, acceptance and value of MDAs to manage NTDs. The previous MDA in these communities took place in the last quarter of 2019, distributing ivermectin as a means to control (in particular) onchocerciasis. Onchocerciasis is a mosquito-driven NTD prevalent in parts of Ghana.

Survey data was collected on electronic devices, using Kobo Toolbox platform, by 47 residents, known as Community-Based Surveillance Volunteers (CBSVs). This is similar to the methods used to distribute medicines during an MDA, albeit data collection that is usually paper-based. The survey was carried out in three hard-to-reach sub-municipalities within Nkwanta South, specifically Alokpatsa (population of 11,028), Brewaniase (14,483), and Tutukpene (15,453). A total of 1,370 responses were received.

One question asked covered whether the individual perceived benefits from the MDA to their household or community, and 98.2% of respondents suggested they did perceive benefits. Only 578 people (44.3%) correctly recalled that the MDA took place more than 2 years ago, but respondents overwhelmingly understood the reasons why such programs are used. For example, 745 people (55.1%) correctly indicated that managing onchocerciasis may reduce the risk of blindness (a known consequence of the infection). Another 596 (44.1%) responded that the MDA would promote general good health. Finally, most respondents were knowledgeable that the program used community volunteers (98.7%) and that the activity was led by the Ghana Health Service (97.2%). The small number of complaints from the survey respondents about the previous MDA overwhelmingly focused on the possibility of being left out (e.g., household missed the exercise or was not registered, *n* = 136, or a medicine shortage, *n* = 31) rather than aspects around their inclusion.

Thus, on the whole, this survey indicated good community recall, knowledge and acceptance of the value of an MDA. Knowing that there is strong evidence that such an intervention is generally well received among these communities is useful for Ghanaian policymakers when planning future health services.

Neighbourhood viewpoints or acceptance prior to the pandemic may be affected or unaffected in the years to come. However, there are concerns that misinformation can become embedded within vulnerable communities, for example around the misuse of ivermectin during the pandemic as a treatment for COVID-19, which has been problematic for public health teams in South America and South-East Asia [6].

Rollouts of health programs such as MDAs can be financially costly and involves significant person-time. Training community volunteers to collect electronic data around knowledge and local viewpoints may be a useful, economical and sustainable approach to inform likely acceptance and be a guide to uptake. It is important to consider community viewpoints on NTDs and other areas of health across other areas of Ghana, plus in other sub-Saharan countries.
